# Plasma calprotectin as an indicator of need of transfer to intensive care in patients with suspected sepsis at the emergency department

**DOI:** 10.1186/s12873-023-00785-y

**Published:** 2023-02-11

**Authors:** Åsa Parke, Christian Unge, David Yu, Jonas Sundén-Cullberg, Kristoffer Strålin

**Affiliations:** 1 Department of Medicine Huddinge, Karolinska Institutet, Stockholm, Sweden; 2 Department of Infectious Diseases, Karolinska University Hospital, Stockholm, Sweden; 3 Department of Medicine, Danderyds Hospital, Stockholm, Sweden; 4 Functional Area of Emergency Medicine, Karolinska University Hospital, Stockholm, Sweden; 5 Division of Clinical Microbiology, Department of Laboratory Medicine, Karolinska Institutet, Stockholm, Sweden; 6 Functional Area of Perioperative Medicine and Intensive Care, Karolinska University Hospital, Stockholm, Sweden

**Keywords:** Biomarkers, P-calprotectin, Sepsis, Sepsis alert, ICU care, Decision-making

## Abstract

**Background:**

Deciding whether to transfer patients with sepsis from the emergency department (ED) to intensive care units (ICUs) is challenging. We hypothesised that the new biomarker plasma calprotectin (p-calprotectin) could be used to aid the selection of patients for intensive care transfer, since it has been shown to be a promising tool for the determination of sepsis severity in critical care.

**Methods:**

This prospective study was performed on consecutive sepsis alert patients in the ED of Karolinska University Hospital Huddinge. The sepsis alert mandates clinical assessment and decisions regarding treatment, disposition, and level of care by physicians from the ED, the Department of Infectious Diseases, and the ICU. Blood sample analysis for C-reactive protein, procalcitonin, neutrophils, and lymphocytes was routinely performed. P-calprotectin was analysed from frozen plasma samples, using a specific turbidimetric assay.

**Results:**

Three-hundred fifty-one patients who triggered the sepsis alert were available for the study. Among 319 patients who were considered to have an infection, 66 patients (26%) were immediately transferred to the ICU or high-dependency unit (HDU), and 253 patients (74%) were transferred to ordinary wards.

Median p-calprotectin was 2.2 mg/L (IQR 1.2–3.9 mg/L) for all patients with infection, it was 3.3 (IQR 1.6–5.2) for those transferred to ICU/HDU and 2.1 (IQR 1.1–3.5) for those transferred to ward units (*p* = 0.0001).

Receiver operating characteristic curve analysis for transfer to the ICU/HDU showed superiority for p-calprotectin compared with procalcitonin and neutrophil–lymphocyte ratio, regarding both all sepsis alert cases and the patients with infection (*p* < 0.001 for all comparisons). The best p-calprotectin cut-off, 4.0 mg/L, showed a sensitivity of 42.5% and specificity of 83% for transfer to the ICU/HDU among patients with infection.

**Conclusions:**

In sepsis alert patients, p-calprotectin was significantly elevated in patients who were subject to immediate ICU/HDU transfer after assessment by a multidisciplinary team. P-calprotectin was superior to traditional biomarkers in predicting the need for transfer to the ICU/HDU.

## Introduction

Sepsis is a common cause of hospital care and admission to intensive care units (ICU) and high-dependency units (HDU). The Global Burden of Disease Study recently found that sepsis accounts for 20% of all global deaths [1]. In western countries, sepsis has been found to have a mortality in the range of 12%–20% [2–5]. The World Health Organisation has therefore highlighted early identification of sepsis as a key global challenge in order to provide for better treatment and improved survival rates [6].

Early recognition and treatment have been shown to improve patient outcomes [7, 8], but sepsis is still a challenge for most hospitals. There is a need for better biomarkers aimed to support clinical decision making at all levels of sepsis care. Procalcitonin (PCT) and C-reactive protein (CRP) are established tools mainly used to distinguish infectious from non-infectious conditions, but their prognostic properties in sepsis care have not been fully clarified [9]. The Surviving Sepsis Campaign recommends PCT as a support for discontinuation of antibiotic therapy [10].

In the early course of sepsis, neutrophils are often activated and lymphocytes are often suppressed [11]. Thus, the neutrophil–lymphocyte ratio (NLR) has been proposed as a useful diagnostic marker of sepsis and has also been shown to have prognostic properties [12, 13].

Calprotectin is a protein present in the cytoplasm of neutrophils and is also expressed on the plasma membrane of monocytes. During the activation of neutrophil granulocytes, calprotectin is released into the circulation. Calprotectin levels have been found to increase in the bloodstream within hours in response to bacteria or endotoxin [14]. Thus, calprotectin in blood samples (plasma or serum) has been proposed as a potential biomarker of sepsis [15–20]. Bartakova et al. found that PCT, CRP, and NLR performed similarly in detecting bacterial sepsis in a hospitalized cohort, whereas the performance of calprotectin was superior [18]. Among patients treated in the ICU, another study found calprotectin to be superior to PCT for the detection of sepsis and for discriminating sepsis from non-septic conditions. With a cut-off of 1.1 mg/L, calprotectin showed a sensitivity of 80% and specificity of 46% for 30-day mortality [20]. Calprotectin has also recently been shown to be a promising biomarker for discrimination of severe and mild COVID-19 [21] and to distinguish infectious respiratory disease from non-infectious respiratory disease [22]. These studies motivate further studies of the prognostic usefulness of calprotectin.

In this study, we aimed to focus on patients with suspected sepsis in the emergency department (ED) and compare the performances of p-calprotectin, PCT, CRP, and NLR in predicting the need for direct transfer to the ICU or HDU as decided by a multidisciplinary team.

## Materials and methods

### Study design

The study was performed at Karolinska University Hospital Huddinge, Stockholm, Sweden, a tertiary care hospital with approximately 700 beds. In the hospital´s ED, which has 75,000 visits per year, all patients are routinely subjected to triage with the Rapid Emergency Triage and Treatment System [23]. Additionally, since September 2017, a sepsis alert has been implemented and is triggered when patients show signs of organ dysfunction combined with symptoms of infection, namely fever, history of fever, or clinical suspicion of infection, as we have described previously [24, 25].

Signs of organ dysfunction are either one of A or B:

A) at least one of the following: oxygen saturation below 90% despite supplemental oxygen administration, respiratory rate greater than 30 per minute, heart rate greater than 130 beats per minute, systolic blood pressure less than 90 mmHg, or Glasgow Coma Scale below 8.

B) blood lactate greater than 3.2 mmol/L combined with at least one of the following: oxygen saturation below 95% on room air, respiratory rate greater than 25 per minute, heart rate greater than 110 beats per minute, altered mental status, and temperature above 38.5 ºC or below 35 ºC.

Patients who trigger the sepsis alert are urgently (within 15 min) assessed bedside by a multidisciplinary team of physicians from the ED, the Department of Infectious Diseases, and the ICU. This is done to identify whether infection is present, optimize clinical care, and aid decision making regarding the level of care—ordinary ward, HDU, or ICU.

The present study was conducted between 27 September 2017, and 31 December 2018. Consecutive patients who triggered the sepsis alert were subjected to structured sampling in the ED, including blood samples for CRP, PCT, neutrophils, lymphocytes, platelets, creatinine, and bilirubin. These samples were immediately analysed at the chemistry laboratory at the hospital, according to routine practice. In addition, plasma samples were collected for subsequent Calprotectin analysis.

Repeated blood samples for CRP, PCT, neutrophils, lymphocytes, and Calprotectin were collected on one of day 2–3 and one of day 5–7, in patients who were still in hospital.

Blood samples for p-calprotectin were collected in a PPT Plasma Preparation Tubes (Becton Dickinson), with immediate centrifugation for 10 min to separate plasma from blood cells, followed by freezing at -20 °C. After a maximum of 3 weeks, the samples were moved to -80 °C. Patients provided written informed consent for study inclusion after sampling. For all patients with written informed consent, plasma samples were thawed and analysed for p-calprotectin, using a specific particle-enhanced turbidimetric assay (Gentian Diagnostics AS; Moss, Norway).

### Data collection

Data regarding demographic characteristics; comorbidity; physiological parameters; clinical, radiological, and microbiological data; and antimicrobial therapy were collected from the patients´ electronic records. The admission sequential organ failure assessment (SOFA) score was calculated from the first physiological parameters registered (in the ambulance or ED) and the first blood samples collected in the ED. A baseline SOFA score was determined by using the latest available SOFA score parameters within the time window between 7 and 90 days prior to admission. If baseline parameters were missing, they were assumed to be normal, as in the development paper for Sepsis-3 [26]. The admission SOFA score minus the baseline SOFA score generated a delta-SOFA score considered to be due to the current infection.

### Definitions of infection and sepsis

Sepsis alert patients were considered to have a *bacterial infection* if they received antibiotic therapy for 4 days or until death or until discharge, in line with definitions suggested by the Centers for Disease Control and Prevention [27, 28]. Patients were considered to have *viral infection* if a viral microorganism was verified by PCR or serology. Patients with bacterial or viral infection according to these definitions were considered to have sepsis if they had a delta-SOFA score of 2 or more on admission [29]. Septic shock was defined according to the Sepsis-3 criteria, i.e., sepsis with vasopressor requirement to maintain a mean arterial pressure of 65 mm Hg or greater and serum lactate level greater than 2 mmol/L [29].

### Primary outcome measures

The primary outcome was direct transfer to an ICU or HDU from the ED, based on the decision of the multidisciplinary team.

### Statistics

SPSS statistical software (version 24) was used for comparison of proportions, using the chi-square test, and between-group comparisons, using the Mann–Whitney U test. STATA statistical software was used for receiver operating characteristic (ROC) curve analysis and comparison between areas under the ROC curves (AUC). A p-value of < 0.05 was considered significant.

Written informed consent was obtained by all study subjects or their next of kins. The Regional Ethics Committee in Stockholm approved the study (reference number 2017/1358–31). The study was conducted according to Swedish regulations.

## Results

### Patient characteristics

**Fig. 1:**
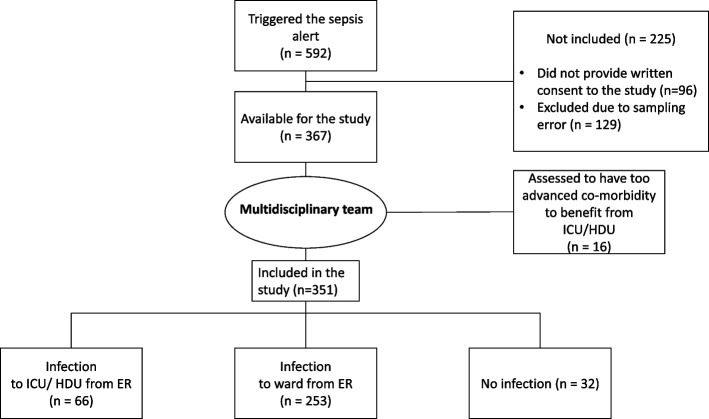
Flow-chart of sepsis alert patients and study patients

In total, 592 patients triggered the sepsis alert during the study period, of which 367 were available for the study. Among these, 319 patients had an infection, and 32 patients had no infection and 16 were too sick to benefit from a higher level of care, after the assessment by the multidisciplinary team (Fig. 1).

Among 319 patients with infections, 312 had bacterial and 7 had viral infections, including influenza virus (*n* = 3), respiratory syncytial virus (*n* = 1), norovirus (*n* = 1), rhinovirus (*n* = 1), and cytomegalovirus infection (*n* = 1). The cytomegalovirus infection was detected by IgM and IgG serology, and the other viral infection cases were detected by PCR.

The patients with infection are described in Table 1, divided into those with or without direct transfer to the ICU/HDU. The groups had similar distributions of age, comorbidities, sources of infection, and 28-day survival rates. They also had similar delta-SOFA scores, although sepsis and septic shock was significantly more common in those transferred directly to the ICU/HDU.

**Table 1:** Characteristics of patients with assumed infection transferred to the ICU/HDU or ward from the ER

Characteristics	Transfer to ICU/HDU (*n* = 66) ^a^	Transfer to ward (*n* = 253) ^a^	*p*-value
**Age, median years (range)**	71.5 (18–94)	73 (19–96)	NS
**Sex, % female**	35	40	NS
**Charlson morbidity score**	5 (0–15)	5 (0–18)	NS
**Comorbidity**	n (%)	n (%)	
Myocardial infarction	5 (7.5)	35 (14)	
Heart failure	14 (21)	44 (17)	
Peripheral vascular disease	7 (10.5)	22 (9)	
Stroke	11 (16.5)	42 (17)	
Dementia	8 (12)	31 (12)	
Chronic obstructive pulmonary disease	7 (10.5)	46 (18)	
Connective tissue disease	3 (4.5)	18 (7)	
Peptic ulcer disease	4 (6)	7 (3)	
Liver disease	5 (7.5)	14 (5.5)	
Diabetes mellitus	16 (24)	59 (23)	
Hemiplegia	1 (1.5)	10 (4)	
Moderate to severe kidney failure	13 (19.5)	54 (21)	
Solid tumour	10 (15)	54 (21)	
Lymphoma	4 (6)	8 (3)	
Leukaemia	5 (7.5)	13 (5)	
HIV/AIDS	0 (0)	1 (0)	
**Source of infection**			
Respiratory tract	22 (33)	104 (41)	0.001
Urogenital	18 (27)	51 (20)	NS
Intra-abdominal	7 (11)	24 (9.5)	NS
Skin/joint/soft tissue/bone	6 (9)	33 (13)	NS
Endocarditis	1 (2)	6 (2)	NS
Other	0 (0)	10 (3)	NS
Unknown	12 (18)	25 (10)	NS
**Delta-SOFA score**	2.5 (0–8)	3 (0–10)	NS
**Sepsis**	57 (86)	204 (81)	< 0.001
**Septic shock**	16 (24)	0 (0)	< 0.001
**ICU/HDU/dead during hospital stay**	66 (100)	13 (5)	< 0.001
**Dead within 28 days**	6 (9)	22 (9)	NS
**Positive blood cultures**	26 (39)	81 (32)	NS
**Blood culture species (most frequent)**			
*Escherichia coli*	11 (44)	20 (26)	
*Streptococcus* spp.	4 (16)	11 (14)	
*Klebsiella* spp.	3 (12)	12 (15)	
*Pseudomonas* spp.	3 (12)	4 (5)	
*Staphylococcus aureus*	1 (4)	6 (8)	
*Streptococcus pneumoniae*	0 (0)	6 (8)	

*ICU* intensive care unit, *HDU* high-dependency unit, *NS* non-significant (> 0.05)

^a^ Data are presented in median, numbers (%), unless otherwise stated

The 32 patients with no infection had a median age of 65 years (range 21–93 years), and 31% (*n* = 10) were female. They had a mean Charlson comorbidity score of 4.0 (range 0–9), a median delta-SOFA score of 3.0 (range 0–9), a 28-day mortality of 3%, and direct transfer to the ICU/HDU was noted in 10 cases (31%). They eventually received the following discharge diagnoses: ketoacidosis (*n* = 5), dehydration (*n* = 4), syncope (*n* = 3), pulmonary embolism (*n* = 3), central chest pain (n = 3), exacerbation of chronic obstructive pulmonary disease (*n* = 2), intoxication (*n* = 2), abdominal pain (*n* = 2), heart failure (*n* = 2), systemic inflammation (*n* = 2), peptic ulcer (*n* = 1), epilepsy (*n* = 1), drug fever (n = 1), headache (*n* = 1), and stress-related disorder (*n* = 1).

The 16 patients who were assessed too sick to benefit from higher level of care hade a median age of 84 years (IQR 75–92), a median delta-SOFA score of 3 (IQR 2–4), and median Charlson comorbidity score of 5.5 (IQR 4–8).

### Performance of p-calprotectin and other biomarkers

**Fig. 2:**
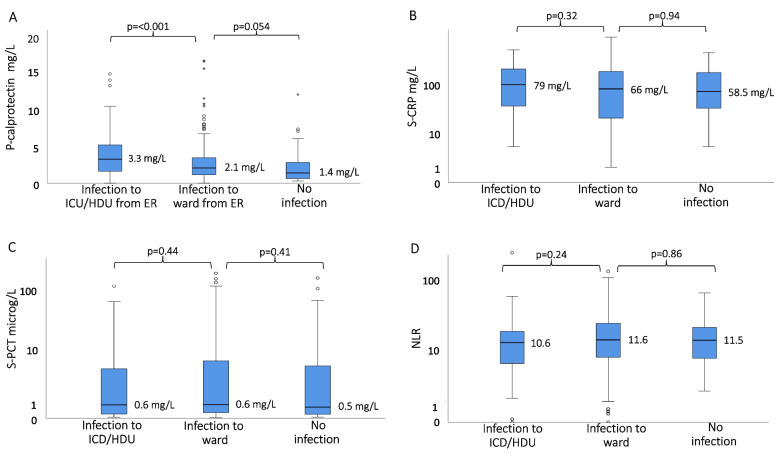
Distribution of p-calprotectin **A**, CRP **B**, procalcitonin PCT; **C** and neutrophil–lymphocyte ratio NLR; **D** in patients with infection transferred to intensive care unit (ICU)/ high-dependency unit (HDU) or ward, and with no infection

**Fig. 3:**
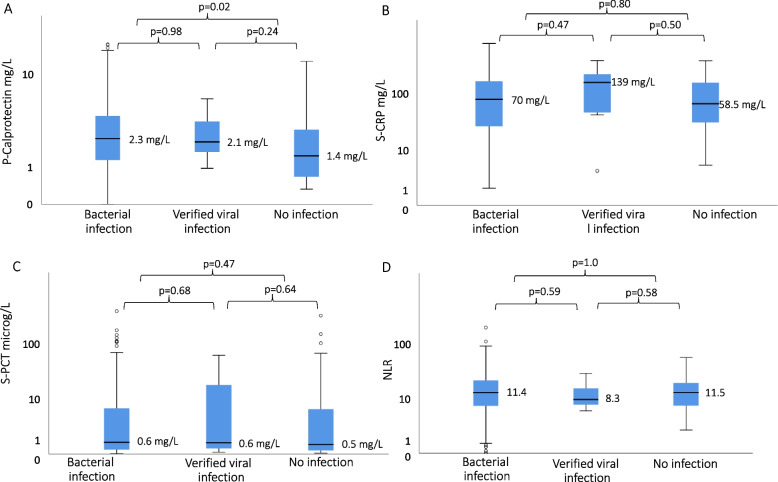
Distribution of p-calprotectin **A**, CRP **B**, procalcitonin PCT; **C** and neutrophil–lymphocyte ratio NLR; **D** in patients with assumed bacterial or viral infection, or no infection

The 351 included sepsis alert patients had the following biomarker results (medians, IQR): p-calprotectin 2.2 mg/L (IQR 1.1–3.8 mg/L), CRP 67 mg/L (IQR 23–146 mg/L), PCT 0.6 µg/L (IQR 0.2–6.0 µg/L), and NLR 11.4 (IQR 6.1–18.7). Figure 2 shows the distribution of p-calprotectin, CRP, PCT, and NLR in patients with infection with and without direct transfer to the ICU/HDU, and in patients without infection. As noted, those transferred to ICU/HDU had significantly higher median p-calprotectin than those transferred to ward units, i.e. median p-calprotectin 3.3 (IQR 1.6–5.2) vs 2.1 (IQR 1.1–3.5; *p* = 0.0001). No similar tendency was noted for PCT, CRP, or NLR. There was no significant difference in the distributions of p-calprotectin, CRP, PCT, and NLR among patients with bacterial infection and viral infection (Fig. 3). Only p-calprotectin could distinguish between bacterial infection and no infection.

Table 2 shows median calprotectin levels for different patient characteristics. P-calprotectin was similar in almost all the different groups analysed and only a few significant differences between groups were detected.

**Table 2:** P-calprotectin values for patients with infection, for different patient characteristics

Characteristics	Calprotectin (mg/L), median (IQR)
**Sex**	
Female (*n* = 124)	2.1 (1.1–3.6)
Male (*n* = 195)	2.4 (1.3–3.9)
**Age, years**	
< 40 (*n* = 15)	2.1 (0.8–3.7)
41–65 (*n* = 91)	2.3 (1.3–4.0)
66–80 (*n* = 134)	2.2 (1.2–3.5)
> 81 (*n* = 79)	2.2 (1.2–3.9)
**Delta-SOFA**	
0–1 (*n* = 58)	1.8 (1.1–3.5)
> 2–4 (*n* = 204)	2.4 (1.3–3.9)
> 4 (*n* = 57)	2.1 (1.2–4.0)
**Source of infection**	
Respiratory tract (*n* = 126)	2.6 (1.5–4.3)
Urogenital (*n* = 69)	2.2 (1.3–3.9)
Intra-abdominal (*n* = 31)	1.7 (0.9–3.3)
Skin/joint/soft tissue (*n* = 39)	2.3 (1.2–3.7)
**Comorbidity (Charlson score)**	
0–1 (*n* = 21)	2.4 (1.3–6.0)
2–5 (*n* = 162)	2.2 (1.1–3.9)
6–10 (*n* = 118)	2.2 (1.2–3.6)
> 10 (*n* = 19)	3.0 (2.0–5.9)
**Sepsis status**	
Sepsis (*n* = 261)	2.3 (1.3–4.9)
Not sepsis (*n* = 58)	1.8 (1.1–3.5)
**Death with in 28 days**	
Dead within 28 days (n = 28)	3.1 (1.2–5.9)
Lived after 28 days (n = 291)	2.2 (1.2–3.8)
**Blood culture status**	
Positive blood culture (107)	2.3 (1.2–3.9)
Negative blood culture (212)	2.2 (1.2–3.8)
**Immunosuppression**^**a**^	
Yes (46)	1.8 (1.2–2.9)
No (273)	2.3 (1.2–3.9)

^a^ Immunosuppression was defined as 10 mg prednisolone or more, or equivalent treatment with other immunosuppressive drugs, or chemotherapy within 28 days

The median p-calprotectin values were similar between sexes, age groups, sources of infection, septic and no-septic patients, and blood cultures with or without bacterial growth. Calprotectin was significantly higher in the two groups with the highest delta-SOFA-values.

**Fig. 4:**
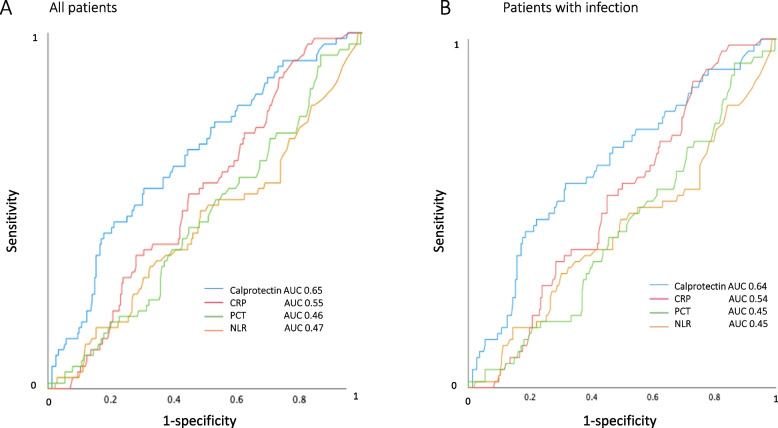
Receiver operating characteristic curve analysis of p-calprotectin, CRP, procalcitonin (PCT) and neutrophil–lymphocyte ratio (NLR) for transferral to intensive care unit (ICU)/ high-dependency unit (HDU) from the emergency department, analysis on all sepsis alert patients (*n* = 351; A) and patients with infection (*n* = 319; B), respectively

ROC and of area under the curve (AUC) analysis for direct transferal to the ICU/HDU were calculated (Fig. 4). When the analysis included all sepsis alert patients and all patients with infections, p-calprotectin had significantly larger AUC than PCT and NLR and tended to have larger AUC than CRP. AUC differences between p-calprotectin and the other biomarkers are shown in Table 3.

**Table 3:** Differences in AUC between p-calprotectin and CRP, PCT, and NLR, respectively

Comparison	All patients		
	*AUC difference*	*95% CI*	*p-value*
*Calprotectin vs CRP*	0.105	0.209–0.005	0.051
*Calprotectin vs PCT*	0.194	0.287–0.078	< 0.001
*Calprotectin vs NLR*	0.178	0.275–0.082	< 0.001
	Patients with infection		
	*AUC difference*	*95% CI*	*p-value*
*Calprotectin vs CRP*	0.101	0.191–0.023	0.08
*Calprotectin vs PCT*	0.19	0.283–0.052	0.002
*Calprotectin vs NLR*	0.187	0.292–0.073	< 0.001

*AUC* area under the curve, *CI* confidence interval

As noted, AUC was significantly higher for calprotectin than for PCT and NLR, regarding both all sepsis alert cases and the patients with infection.

Sensitivities, specificities, and predictive values were calculated for different p-calprotectin concentrations for direct transfer to the ICU/HDU (Table 4).

**Table 4:** Diagnostic performance of plasma-calprotectin cut-offs for transfer to ICU/HD among patients with infection

Calprotectin cut-off value (mg/L)	Sensitivity ^a^	Specificity ^b^	Positive predictive value ^c^	Negative predictive value ^d^
2	70 (46/66)	52 (131/253)	25 (46/185)	86 (122/142)
2.5	61 (40/66)	66 (155/253)	29 (40/138)	86 (155/181)
3	53 (35/66)	70 (177/253)	31.5 (35/111)	85 (177/208)
3.5	48.5 (32/66)	76 (192/253)	34 (32/93)	86 (192/226)
4	42.5 (28/66)	83 (209/253)	39 (28/72)	85 (209/247)
4.5	38 (25/66)	84 (212/253)	38 (25/66)	84 (212/253)

^a^ % (No. of true-positive cases/all cases transferred to ICU/HDU)

^b^ % (No. of true-negative cases/all cases not transferred to ICU/HDU)

^c^ % (No. of true-positive cases/all cases above cut-off)

^d^ % (No. of true-negative cases/all cases under cut-off)

Thirteen patients initially transferred from ER to ward units, were later (12–72 h from admission) transferred from the ward units to ICU/HDU. Their ER median p-calprotectin was 4.1 (IQR 1.1–6.0).

**Fig. 5:**
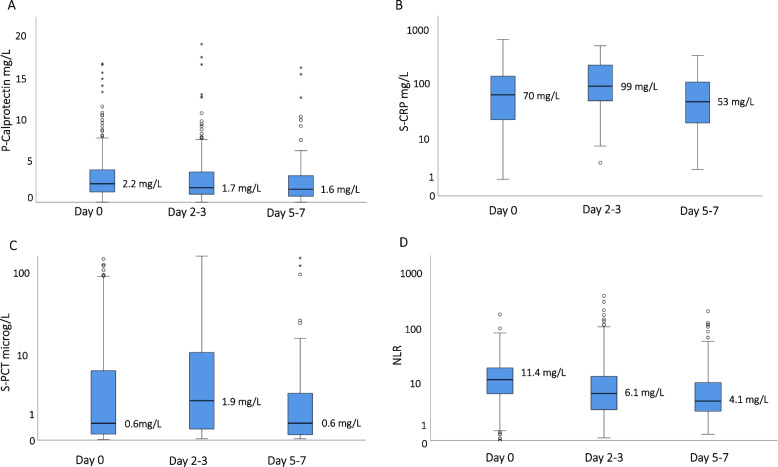
Distribution of p-calprotectin **A**, CRP **B**, procalcitonin PCT **C** and neutrophil–lymphocyte ratio NLR **D** in blood samples collected on hospital days 0, 2–3, and 5–7

Among 319 patients with infection, 223 (70%) patients had day 2–3 samples and 176 (55%) had a day 5–7 samples for p-calprotectin collected. The corresponding figures were 198 (62%), 151 (47%) for CRP (*n* = 316); 198 (62%), 150 (47%) for PTC (*n* = 297); and182 (57%), 138 (43%) for NLR (*n* = 288). Figure 5 shows dynamic changes of p-calprotectin, CRP, PCT, and NLR over time in patients with repeated samples. Calprotectin was almost unchanged from day 0 to days 2–3, and after that it decreased. CRP and PCT continued to rise until days 5–7. NLR was decreasing from days 2–3.

## Discussion

In this study of sepsis-alert patients, we found that the novel biomarker p-calprotectin was elevated in patients with established infection and was superior to CRP, PCT, and NLR in detecting patients who were transferred directly to the ICU/HDU care among patients triggering a sepsis alert system and assessed by a multidisciplinary team.

P-calprotectin has been shown to be a potential biomarker for discriminating sepsis patients from other patients with infection [16–19]. Due to the heterogeneity of patients with sepsis, previous studies have focused on the role of calprotectin in multi-organ failure [30]. We wanted to study p-calprotectin in a well-characterized cohort of patients with clinical suspicion of sepsis who were already in the ED and evaluate whether p-calprotectin may be of value in the early assessment of sepsis. We found that patients with infection who the multidisciplinary team in the ED decided to transfer to the ICU/HDU had significantly higher p-calprotectin than those who were transferred to ordinary wards (Fig. 2). This was not explained by greater comorbidity (as measured by the Charlson comorbidity score) or disease severity (as measured by SOFA score increase) at presentation. There was no significant difference in delta-SOFA score between those transferred to the ICU/HDU and those transferred to wards. This illustrates the difficulties of using only the SOFA score for severity assessment. In the present study, the sepsis alert patients were assessed by a multidisciplinary team who decided where to transfer the patient.

Our study showed that CRP, PCT, and NLR could not discriminate between those who were directly transferred to the ICU/HDU and those who were not. However, ROC curve analysis indicated that p-calprotectin has greater potential to be useful in assessing the need for transfer to the ICU/HDU, as it was found to be superior to CRP, PCT, and NLR regarding this issue (Fig. 4, Table 4). As shown in Fig. 4, p-calprotectin had an AUC of 0.65 for direct transfer to ICU/HDU care. Accordingly, Larsson et al. recently studied calprotectin in ICU patients and found an AUC of 0.67 for discrimination between sepsis and non-sepsis in the ICU, and 0.64 for prediction of ICU death [20]. However, when looking at sensitivities, specificities, and predictive values, we found the p-calprotectin value of 4.0 mg/L to be the best cut-off for predicting the need for ICU/HDU care, with a sensitivity of 42.5% and specificity of 83%. Larsson et al. found the best calprotectin cut-off to be 1.3 mg/L for discrimination between sepsis and non-sepsis, with a sensitivity of 81% and specificity of 56%, and 1.1 mg/L for prediction of ICU death, with a sensitivity of 80% and specificity of 46% [20]. This difference might be related to patient selection or the assay for calprotectin used. The present study included mainly patients with community-acquired infection with a high rate of sepsis, in contrast to the other studies, which might have led to a different cut-off in our results.

P-calprotectin and the other biomarkers did not differ considerably in performance based on sex, age, co-morbidity, and source of infection, and there was no clear difference in p-calprotectin levels between bacterial and viral infection. However, there were very few viral infections, and this made the numbers difficult to interpret. This was also the case for CRP, PCT, and NLR, and may be due to the small number of patients with viral infection in our study. Also, to be noted, the calprotectin distribution was not related to blood culture positivity/negativity (Table 2), but blood cultures are only positive in 30%–40% of sepsis patients, even though these patients are diagnosed as having bacterial infection [31]. Faecal calprotectin has been shown to be affected by immunosuppression [32], but we found no difference in p-calprotectin levels in patients with or without immunosuppression.

Patients initially transferred to ward units, who were subsequently transferred from ward to ICU/HDU within 12–72 h from presentation, had initial high p-calprotectin levels. This finding indicates that a stable patient who does not require ICU/HDU transferral from ED, but who has a high p-calprotectin level, may have an increased risk of subsequent requirement of ICU/HDU transferral. The prognostic properties of p-calprotectin should be further studied.

Looking at the ED sample and day 2–3 sample, p-calprotectin and NLR showed decreasing levels, whereas CRP and PCT increased (Fig. 5). This may reflect different inherent kinetics of the biomarkers. Our findings differ from those of Bartakova et al., and Simm et al. [16, 18]. In Bartakova et al.´s study, all biomarkers had the same dynamics as those we found for calprotectin and NLR [18]. However, in the study by Simm et al., only CRP had the same pattern as calprotectin [16]. This may reflect different timing of sampling and a more heterogeneous group of patients in our study compared with the study by Simm et al. Since calprotectin increases early during infection, it could perhaps be an interesting biomarker in pre-hospital care settings. Further studies will be needed to define its exact role.

This study has several strengths. First, our study cohort was well characterized and clinically stratified for suspected sepsis, since all patients had triggered the sepsis alert in the ED. Thus, all patients, even those who did not have any infection, had high triage scores due to severe illness. Second, all samples were collected upon presentation in the ED, before initiation of treatment. Therefore, confounding effects of treatment were minimized.

The study also has limitations. The study did not include sepsis alert patients without written informed consent, including 35 patients who died during their hospitalisation. Possibly, inclusion of such patients would have changed the results.

## Conclusion

In sepsis alert patients, p-calprotectin was elevated in those who a multidisciplinary team decided to transfer immediately to the ICU/HDU. P-calprotectin was superior to traditional biomarkers in predicting the need for ICU/HDU care. Thus, adding the results of the present study to previous studies, p-calprotectin appears to be a useful biomarker in the management of sepsis.

## Data Availability

The datasets used and/or analyzed during the current study are available from the corresponding author on reasonable request.

## Abbreviations

PCT: Procalcitonin
CRP: C-reactive protein
NLR: Neutrophil-lymphocyte ratio
ICU: Intensive care unit,
ED: Emergency department
HDU: High-dependency unit
SOFA: Sequential organ failure assessment
AUC: Area under the curve
